# An oldest‐old non‐small cell lung cancer patient with abscopal effect in a single lesion

**DOI:** 10.1111/1759-7714.14551

**Published:** 2022-06-27

**Authors:** Tadashi Sakaguchi, Kentaro Ito, Kentaro Fujiwara, Yoichi Nishii, Satoru Ochiai, Yoshihito Nomoto, Osamu Hataji

**Affiliations:** ^1^ Matsusaka Municipal Hospital Respiratory Center Matsusaka Japan; ^2^ Department of Radiation Oncology Japanese Red Cross Ise Hospital Ise Japan; ^3^ Department of Radiology Mie University School of Medicine Tsu Japan

**Keywords:** abscopal effect, case report, non‐small cell lung cancer, oldest‐old, palliative radiation

## Abstract

The abscopal effect without concomitant immunotherapy is a rare event, including among cases of lung cancer. Furthermore, the occurrence of limited abscopal effect for only a single lesion in the metastatic organ consistent with the irradiated organ would be an even more rare event. A 94‐year‐old man was diagnosed with advanced lung cancer with osteolytic bone metastases in his right iliac bone, and the right side of his axial vertebrae. After palliative radiation therapy to the right iliac lesion for pain relief without other anticancer therapy, the axial vertebral osteolytic lesion disappeared despite no reduction in the other lesions. This case furthers our understanding of the pathogenesis of the abscopal effect.

## Graphical abstract

An oldest‐old non‐small cell lung cancer patient with bone metastases in his iliac bone and axial vertebrae. After palliative radiation therapy to his iliac lesion for pain relief, the axial vertebral osteolytic lesion disappeared despite no reduction of the other lesions. The abscopal effect is a rare event; furthermore, an occurrence of limited abscopal effect for only a single lesion in the metastatic organ consistent with the irradiated organ would be an even more rare event.

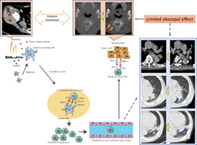

## INTRODUCTION

The abscopal (ab− “position away from” and scopus “target”) effect is a rare event of tumor regression at a site distant from an irradiated field. It was first described by Mole in 1953 and has since been observed in different types of cancers, especially in immunogenic tumors, such as renal cell carcinoma, melanoma, and lymphoma, after radiotherapy directed at the primary tumor or metastases.^1^, ^2^

A recent review of the abscopal effect in patients with advanced lung cancer showed seven cases that experienced the abscopal effect without immune therapy;^3^ however, few cases have shown the abscopal effect only for a limited lesion.

## CASE REPORT

**FIGURE 1:**
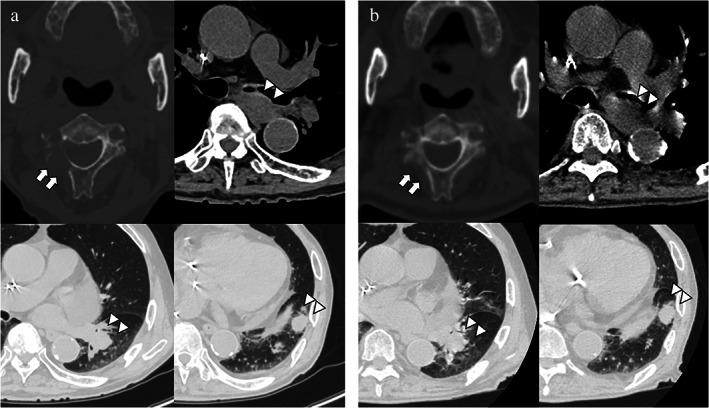
Abscopal effect for only a single lesion. (a) Computed tomography (CT) findings before radiation therapy to the right iliac bone metastasis. (b) CT findings after the radiation therapy. The white arrows show abscopal effect of axial vertebral bone metastasis and white arrowheads indicate other tumor lesions without change in tumor size

A 94‐year‐old man was referred to our hospital due to a suspicion of lung cancer and metastatic bone tumors. His medical history included chronic kidney disease, and sick sinus syndrome that required pacemaker implantation. He had right inguinal pain, and his CT scan revealed osteolytic bone metastases in his right iliac bone and the right side of his axial vertebrae, two pulmonary lesions in his left lower lobe, and mediastinal lymph node metastasis between his left main bronchus and esophagus (Figure 1a). Squamous cell carcinoma was detected by percutaneous CT‐guided needle biopsy of the osteolytic right iliac lesion, and he was clinically diagnosed with advanced squamous cell lung carcinoma with a high tumor proportion score (TPS). He did not request chemotherapy, only palliative treatment, due to his old age and poor performance status (ECOG PS 3). We consulted the department of radiation oncology for palliative radiation therapy to both his right iliac lesion for relief of his inguinal pain, and his axial vertebrae lesion for prevention of nerve invasion. He was to receive radiation therapy to his right iliac lesion first, followed by the axial vertebrae lesion, because he could not hold his posture long enough to treat both lesions simultaneously. Seven days after the completion of the radiation therapy to his right iliac lesion, at a total dose of 30 Gray (Gy) in 10 fractions, a CT scan for planning the radiation therapy to his axial vertebrae lesion was performed and revealed that the osteolytic lesion had disappeared (Figure 1b). There was no change, however, in the size of other nonirradiated pulmonary and mediastinal lymph lesions, and newly hepatic metastases were observed. Subsequently, the mediastinal lymph lesion was irradiated at a total dose of 45 Gy in 15 fractions due to stenosis symptoms in his airway and esophagus. After the irradiation therapy, the improved osteolytic lesion in his axial vertebrae passed without re‐aggravation, and the patient was transferred to the palliative care ward of another hospital.

## DISCUSSION

**FIGURE 2:**
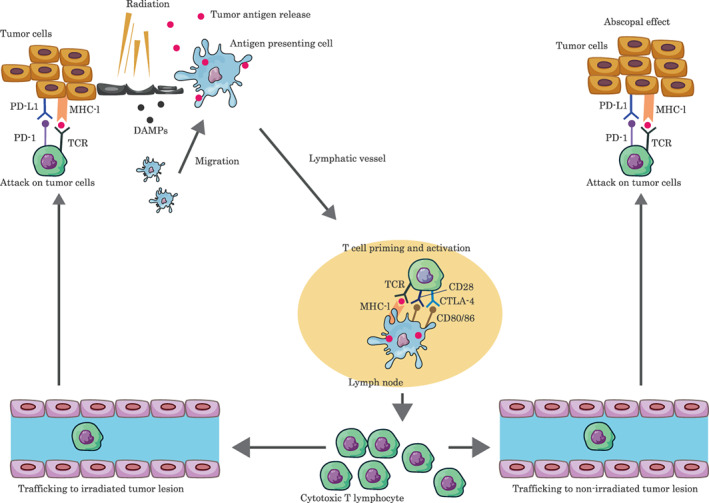
Mechanism of the abscopal effect. Immunogenic cell death is induced by local radiation therapy releasing tumor antigens, which stimulate the immune system of the host systemically and trigger the regression of not only irradiated cancer cells but also distant nonirradiated cancer cells. CTLA‐4, cytotoxic T‐lymphocyte‐associated antigen 4; DAMP, damage‐associated molecular pattern; MHC, major histocompatibility complex; PD‐1, programmed cell death protein 1; PD‐L1, programmed death ligand 1; TCR, T cell receptor

Although case reports of the abscopal effect in combination with immunotherapy for patients with advanced lung cancer are increasing due to the synergic effect of the immune mechanism,^3^ an incidence of pure abscopal effect without the combination of immunotherapy is a rare phenomenon. Furthermore, case reports of the abscopal effect for only a localized lesion, as in this case, are extremely rare. Interestingly, the abscopal effect in this case was shown in another bone metastasis only after a bone metastasis was irradiated. Although the exact molecular mechanisms for the abscopal effect are yet to be established, some reviews explain the mechanism in the following manner,^3^, ^4^, ^5^ which is also summarized in Figure 2. Immunogenic cell death is induced by local radiation therapy releasing tumor antigens, and the immune system can recognize damaged cells through the identification of specific molecules known as damage‐associated molecular patterns (DAMPs). Some DAMPs are able to trigger the maturation of antigen presenting cells such as dendritic cells, which leads to the proliferation and activation of CD8^+^ T cells. The activation of CD8^+^ T cells requires the cross‐presentation of exogenous tumor antigens on major histocompatibility complex (MHC) class I molecules. Naive CD8^+^ T cells receive the antigen‐specific signal through the T‐cell receptor (TCR) and costimulatory signals such as CD80 and CD86 through CD28. Tumor antigen‐specific CD8^+^ T cells proliferate and differentiate into cytotoxic effector T cells (CTLs) that migrate from the lymph nodes to the tumor sites (primary tumor and nonirradiated tumor metastases) in order to exercise their effect of killing tumor cells. One hypothesis about why the abscopal effect after bone irradiation was limited to another bone metastasis in this case is that the released tumor antigens recognized by the immune system may be some of relevant substance for metastasis to bone, which cancer cells acquire in the process of metastasis to bone. Additionally, as to the reason of such insufficient abscopal effect, decreased immunity due to aging, high expression of PD‐L1 of the tumor, and low dose per fraction irradiation might be related. To the best of our knowledge, although there have been no reports evaluating the relationship between aging and the abscopal effect in detail, one review described that the mean age mentioned in the case reports was 57.5 years with a standard deviation of 15.1 years,^6^ and that reports of abscopal effects in oldest‐old patients were extremely rare.^7^ As for the relationship between immunity and aging, a reduction in T cell diversity is seen in older individuals and linked with increased susceptibility to infection, autoimmune disease, and cancer. Furthermore, age‐related regression of the thymus is one of the most dramatic and ubiquitous changes seen in the aging immune system.^8^ For irradiation dose and fractionation, the Japanese guidelines for treatment of bone metastasis recommend multifractionated radiation, such as 30 Gy in 10 fractions or 20 Gy in five fractions, and single‐dose irradiation such as 8 Gy.^9^ Although the optimal dose and fractionation schedules for radiation‐induced immune priming are unclear, growing preclinical evidence indicates that high‐dose irradiation per fraction can promote the release of DAMPs that lead to the recruitment of immune cells, and induce a systemic response against tumor antigens that protects against local disease relapse and also mediates distant antineoplastic effects.^10^ Additionally, in a clinical trial, the out‐of‐field overall response rates of stereotactic body RT (50 Gy in 4 fractions) is better than that of traditional hypofractionated RT (45 Gy in 15 daily fractions), although this was in combination with immunotherapy.^11^ Further research is needed to establish strategies for radiation therapy to maximize antitumor immunity effects in clinical settings.

## CONFLICT OF INTEREST

K. Ito has received speaker fees as honoraria from Eli Lilly Japan, Chugai, AstraZeneca, MSD, Boehringer Ingelheim Japan, Ono, and Pfizer Japan. O. Hataji received grant funding and speaker fees as honoraria from GlaxoSmithKline, Daiichi Sankyo, Bayer, Novartis Pharma, AstraZeneca, and Boehringer Ingelheim Japan. The remaining authors declare no conflict of interest.
